# Initial lactate levels and 14-day mortality in ischemic stroke: A retrospective cohort study using the MIMIC-IV database

**DOI:** 10.1097/MD.0000000000050308

**Published:** 2026-08-21

**Authors:** YuHao Zhu, Xinyuan Liu, Hanghang Song

**Affiliations:** a School of Basic Medical Sciences, Heilongjiang University of Traditional Chinese Medicine, Harbin, Heilongjiang, China; b Qingdao University, Qingdao, Shandong, China.

**Keywords:** ischemic stroke, lactate, Medical Information Mart for Intensive Care IV database, potassium ion, short-term mortality

## Abstract

Ischemic stroke is a leading cause of death and disability worldwide, and early prognostic assessment is crucial for optimizing patient management. Lactate is an important biomarker reflecting tissue hypoxia and metabolic disorders, and its relationship with short-term mortality risk has not been fully clarified. This study aimed to explore the association between early lactate levels on admission and the risk of mortality within 14 days in patients with ischemic stroke. This study was a retrospective cohort study based on the Medical Information Mart for Intensive Care IV database. Cases with high-risk underlying diseases such as hypertension and diabetes were excluded. Univariate and multivariate logistic regression analyses were used to evaluate the relationship between lactate levels and short-term mortality risk, and the receiver operating characteristic curve was used to analyze the predictive ability of lactate and other biochemical indicators. In addition, subgroup analysis was performed to explore the interaction of biochemical indicators in patient groups with different mortality risks. A total of 157 patients with ischemic stroke were included, comprising 107 in the survival group and 50 in the death group. Univariate analysis showed that lactate levels were significantly associated with 14-day mortality (*P* < .05), but multivariable logistic regression analysis revealed that lactate levels were not independent risk factors (*P* > .05). Potassium and sodium ion levels were independent risk factors for short-term mortality (*P* < .05). Receiver operating characteristic analysis showed that the area under the curve of lactate, potassium, and sodium ions was between 0.60 and 0.70, indicating limited predictive ability. Subgroup analysis found that lactate was associated with sodium, chloride, and blood glucose levels in the survival group, while lactate was associated with calcium, sodium, and chloride ions in the non-survival group, suggesting potential differences in lactate–electrolyte interactions between groups in different populations. Initial lactate levels were associated with 14-day mortality in univariate analysis but were not an independent predictor after multivariable adjustment. Sodium and potassium levels were independently associated with short-term mortality in patients with ischemic stroke. The discriminative performance of lactate alone was limited (area under the curve = 0.637, 95% confidence interval: 0.546–0.728).

## 1. Introduction

Ischemic stroke is the second leading cause of death worldwide and one of the main diseases that cause death and disability in modern humans. Its morbidity and mortality rates remain high.^[1]^ The pathogenesis of stroke includes a series of complex physiological and pathological reactions such as cellular excitotoxicity, oxidative stress, neuroinflammation, and metabolic disorders, which ultimately lead to brain tissue damage and dysfunction.^[2]^ In this process, lactate, as an important metabolite of tissue hypoxia and metabolic imbalance, is one of the main energy sources for neuronal mitochondrial oxidative phosphorylation and can quickly reflect the anaerobic metabolic state. However, the value of lactate levels in the short-term prognosis of patients with ischemic stroke has not been fully clarified. In ischemic stroke, lactate levels on the first day of hospitalization may be related to the severity of brain tissue ischemia, enhanced anaerobic metabolism, and systemic inflammatory state.^[3,4]^ Some recent studies have shown that high lactate levels are associated with poor prognosis in patients,^[5,6]^ but its short-term prognosis in stroke patients needs further exploration. Initial lactate levels may provide an early and convenient means of risk assessment for identifying high-risk patients and help optimize treatment strategies. However, there is still insufficient evidence on the relationship between lactate levels and the 14-day short-term mortality risk of stroke patients. Early risk identification is crucial for improving the clinical prognosis of patients with ischemic stroke. Traditional stroke prognostic assessment tools such as the National Institute of Health Severity Score or imaging examinations have certain limitations, while lactate, as an easily measurable biomarker, can provide additional prognostic information.^[7]^ By analyzing the Medical Information Mart for Intensive Care IV (MIMIC-IV) database, this study aims to evaluate the role of lactate levels on the first day of hospitalization in predicting the risk of death within 14 days in patients with ischemic stroke and to explore the clinical feasibility of lactate levels as an early risk assessment tool in order to provide a scientific basis for stroke management. Through this study, we hope to further clarify the importance of lactate in the pathophysiology of stroke and verify its prognostic value as a biomarker, thereby laying the foundation for early intervention and individualized treatment of ischemic stroke.

Several recent studies have investigated the relationship between lactate-related metabolic indicators and outcomes in patients with ischemic stroke.^[8–10]^ Elevated lactate levels may reflect tissue hypoxia, impaired perfusion, and metabolic stress during the acute phase of stroke, and some studies have suggested that lactate or lactate-related indices may be associated with increased mortality risk. However, the prognostic significance of early lactate levels remains controversial, and evidence regarding their role in predicting short-term mortality in ischemic stroke patients is still limited. Early mortality following stroke is often related to the acute pathophysiological response and early complications during hospitalization. Previous studies have reported that a considerable proportion of deaths occur within the first days to weeks after stroke onset, highlighting the importance of early outcome assessment.^[11–13]^ Therefore, the present study selected 14-day mortality as an indicator of short-term prognosis.

## 2. Methods

### 2.1. Data source

This was a retrospective cohort study based on the MIMIC-IV 3.0 database. The MIMIC-IV database is a publicly accessible critical care database known for its large amount of clinical data on patients treated in intensive care units (ICUs) between 2008 and 2022. It is recognized as one of the largest and most frequently used databases in the field of critical care. It provides an important resource for research and analysis purposes. With its extensive ICU-related data, MIMIC-IV has become a valuable tool for investigating critical care outcomes, predictive modeling, clinical decision support, and other research areas. Permission to use the MIMIC-IV database in this study was obtained from the Institutional Review Boards of the Massachusetts Institute of Technology and Beth Israel Deaconess Medical Center Boston, MA).

### 2.2. Ethical considerations and data privacy

The present study was conducted using the publicly available MIMIC-IV database. The MIMIC-IV database has received ethical approval from the Institutional Review Boards of the Massachusetts Institute of Technology and Beth Israel Deaconess Medical Center (Boston, MA). Access to the database was granted after the completion of the Collaborative Institutional Training Initiative “Data or Specimens Only Research” course (Record ID: 63549220). All patient data in the MIMIC-IV database are de-identified to protect patient privacy. Therefore, the requirement for informed consent was waived by the Institutional Review Board.

### 2.3. Study population, variable extraction, and outcomes

**Figure 1.**
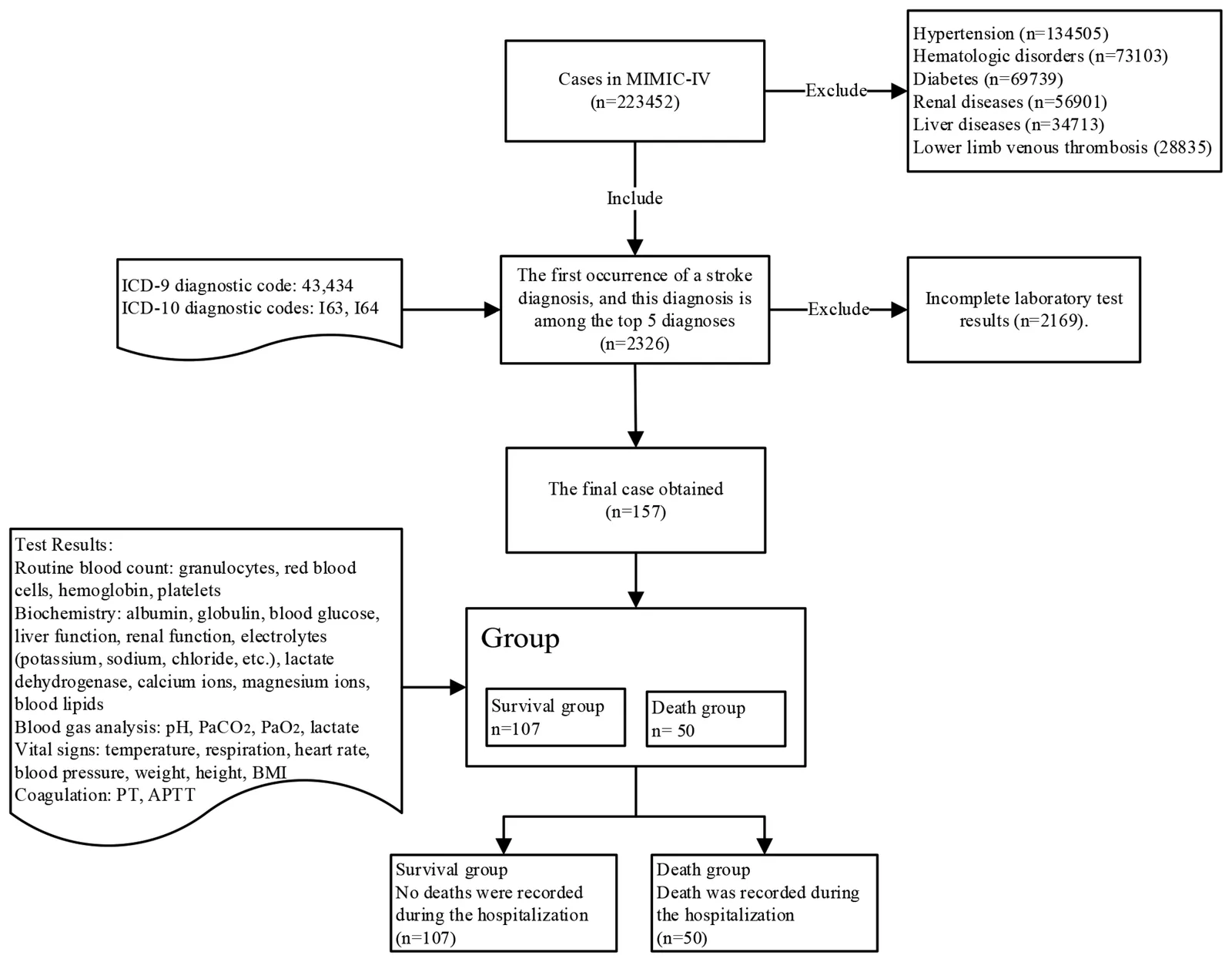
Flowchart of case extraction. APTT = activated partial thromboplastin time, BMI = body mass index, ICD = International Classification of Diseases, n = number of cases, PT = prothrombin time.

Inclusion criteria for the study population: Patients diagnosed with ischemic stroke were identified using International Classification of Diseases (ICD)-9 code 434 and ICD-10 codes I63 and I64. Stroke cases were identified using ICD codes (ICD-9 diagnostic code: 434; ICD-10 diagnostic codes: I63, I64). Exclusion criteria were established to reduce potential confounding effects on lactate metabolism and to improve the internal validity of the study. Patients with major comorbidities, including hypertension, diabetes, liver disease, renal disease, hematological disorders, and lower extremity venous thrombosis, were excluded. While this approach helps isolate the association between lactate levels and short-term mortality, it may limit the generalizability of the findings, which is acknowledged in the Discussion. Exclusion criteria: Cases with hypertension, diabetes, hematological diseases, kidney disease, liver disease, or lower extremity venous thrombosis, and those without blood test records for lactate, creatine kinase (CK), calcium ions, and albumin within 24 hours of admission. The specific data cleaning steps are shown in Figure 1.

### 2.4. Variable extraction and outcomes

Variable extraction: the variables extracted in this study mainly include patient age, gender, height, admission weight, vital signs on the first day of admission, and blood tests on the first day of admission (including blood routine, liver function, renal function, coagulation function, and arterial blood gas analysis), as well as the Glasgow Coma Scale (GCS) score on the first day of admission. Details of all variables are shown in Table 1. Outcome variables: survival and death outcomes of patients within 14 days of admission.

**Table 1:** Detailed description of indicators extracted from the MIMIC-IV database.

Item	Composition
Demographics	Age, gender, BMI, height, weight
Disease severity score	GCS
Vital signs	SBP, DBP, MAP, body temperature, heart rate, respiratory rate, SO_2_
Laboratory tests	
Complete blood test	White blood cells, red blood cells, hemoglobin, platelets
Blood biochemistry test	Calcium ion, sodium ion, potassium ion, chloride ion, blood sugar
Liver function	ALT, AST, ALP, albumin
Damage markers	CK, CKMB
Arterial blood gas analysis	Lactate, PaO_2_, PaCO_2_, pH, SO_2_, anion gap
Coagulation	PT, APTT, INR
Clinical prognosis	There is a death record within 14 days, and there is no death record within 14 days

ALP = alkaline phosphatase, ALT = alanine aminotransferase, APTT = activated partial thromboplastin time, AST = aspartate aminotransferase, BMI = body mass index, CK = creatine kinase, CKMB = creatine kinase-myocardial band, DBP = diastolic blood pressure, GCS = Glasgow Coma Scale, INR = International Normalized Ratio, MAP = mean arterial pressure, MIMIC-IV = Medical Information Mart for Intensive Care IV, PaCO_2_ = Partial pressure of arterial carbon dioxide, PaO_2_ = partial pressure of arterial oxygen, pH = potential of hydrogen, PT = prothrombin time, SO_2_ = oxygen saturation, SBP = systolic blood pressure.

In data processing, laboratory variables were summarized using the average of the maximum and minimum values within the first 24 hours. This approach was adopted to capture both peak abnormalities and baseline physiological status, thereby better reflecting overall physiological fluctuations while reducing the influence of transient measurement variability.The minimum GCS value was used to represent the severity of the patient’s condition when he was admitted to the hospital.

### 2.5. Statistical analysis

Continuous variables are expressed as median (interquartile range). Group comparisons were performed using the Mann–Whitney *U* test. Categorical variables were compared using the chi-square test. *P* < .05 was considered statistically significant. Variables with clinical relevance and those showing associations in univariate analysis were considered candidate variables for multivariable logistic regression analysis. A nomogram was established and the calibration curve model was verified using R language. Receiver operating characteristic curve (ROC) analysis was used to analyze frequently mentioned indicators such as calcium, potassium, and lactic acid in previous stroke patient surveys. The outcome variable was set as death, and the results were presented as area under the curve (AUC) and 95% confidence interval (CI). In subgroup analysis, Pearson correlation analysis was performed between laboratory test indicators and exploratory indicators of survival and death groups to observe potential interactions within subgroups. All statistical analyses were conducted using Statistical Package for the Social Sciences 27 (IBM Corp.). Descriptive statistics and univariate analysis only used observed data. Multiple imputation was used to supplement missing data in regression analysis. Missing covariate data were imputed using multiple imputation by chained equations, and the pooled results were used in the regression analysis. Details of missing values have been marked in the table.

## 3. Results

### 3.1. Demographic characteristics and clinical baseline

Due to missing laboratory measurements for some patients, the sample size varied slightly across different variables. A total of 157 patients were included in this study, including 107 in the survival group and 50 in the death group. There were 62 males and 65 females. The median age of all patients was 65.25 (53.11–79.55) years. There was no statistical difference in gender, age, weight, height, body mass index, GCS score, and vital signs (blood pressure, body temperature, heart rate, respiratory rate, and blood oxygen saturation), as shown in Table 2.

**Table 2:** Comparison of demographics and clinical baseline between the survival group and the death group.

	Survival Group (n = 107)	Death Group (n = 50)	Statistics	*P*
Demographics				
Female	42	23	*χ*^2^ = 0.640	.424
Male	65	27		
Age	65.18 (52.40–77.80)	65.77 (54.23–80.36)	*z* = −0.482	.630
Weight	74.70 (64.90–97.80)	76.17 (60.22–90.85)	*z* = −1.024	.306
Height	170.00 (163.00–178.00)	168.00 (160.00–175.00)	*z* = −0.984	.325
BMI	26.48 (22.55–31.88)	26.23 (21.90–31.78)	*z* = −0.198	.843
Severity of illness				
GCS	15.00 (13.00–15.00)	15.00 (14.00–15.00)	*z* = −1.538	.124
Vital signs				
SBP	119.81 (108.89–133.65)	119.67 (105.78–131.69)	*z* = −0.695	.487
DBP	65.23 (58.78–73.51)	65.45 (57.83–72.68)	*z* = −0.062	.950
MAP	81.88 (75.01–90.25)	83.82 (74.70–89.11)	*z* = −0.002	.998
Temperature	37.05 (36.64–37.34)	37.02 (36.61–37.34)	*z* = −0.461	0.645
Heart rate	83.61 (72.48–92.96)	84.22 (72.93–100.07)	*z* = −0.034	0.973
Respiratory rate	19.28 (17.20–21.47)	20.04 (17.04–24.39)	*z* = −1.098	0.272
SO_2_‡	97.00 (95.00–98.00)	97.00 (95.75–98.25)	*z* = −0.783	0.434

Due to missing laboratory measurements in some patients in the MIMIC-IV database, the number of observations varies slightly across different variables.

BMI = body mass index, DBP = diastolic blood pressure, GCS = Glasgow Coma Scale, MAP = mean arterial pressure, MIMIC-IV = Medical Information Mart for Intensive Care IV, n = number of individuals, SO_2_ = oxygen saturation, SBP = systolic blood pressure.

* Survival case = 75, death case = 38.

† Survival cases = 106; death cases = 43.

‡ Survival cases = 40; death cases = 24.

### 3.2. Laboratory tests

Laboratory tests (leukocytes, hemoglobin, platelets, calcium ions, venous and arterial blood glucose, alkaline phosphatase, albumin, CK, partial pressure of arterial carbon dioxide, potential of hydrogen, anion gap, prothrombin time, activated partial thromboplastin time, International Normalized Ratio) showed no statistical difference between the survival group and the death group (*P* > .05), while sodium ions, potassium ions, chloride ions, alanine aminotransferase, aspartate aminotransferase, CK-myocardial band, lactate, and bicarbonate showed a statistical difference between the survival group and the death group (*P* < .05). Among the exploratory indicators, only indicators with no missing samples were included in our study, and the calculation formula for each indicator was: $exploratory\;indicators=\frac{Laboratory\;tests}{lactate}$. Several exploratory ratios, including the arterial glucose-to-lactate ratio, venous glucose-to-lactate ratio, albumin-to-lactate ratio, potassium-to-lactate ratio, calcium-to-lactate ratio, and chloride-to-lactate ratio, showed statistical differences between the survival and death groups (*P* < .05). These findings should be interpreted as exploratory and hypothesis-generating. See Table 3 for details.

**Table 3:** Comparison of general information, laboratory test indicators and exploratory indicators.

	Survival Group (n = 107)	Death group (n = 50)	Statistics	*P*
Laboratory tests				
Leukocyte	12.45 (9.10–16.23)	14.27 (11.00–17.60)	*z* = −1.891	.059
Hemoglobin	11.55 (9.15–13.20)	11.68 (9.85–12.70)	*z* = −0.002	.998
Platelets	198.00 (148.00–272.25)	210.25 (148.50–262.00)	*z* = −0.224	.823
Calcium	8.20 (7.80–8.70)	8.48 (8.10–8.75)	*z* = −1.679	.093
Sodium ion	139.50 (136.00–142.00)	140.75 (138.50–145.50)	*z* = −2.609	.009
Potassium	4.20 (3.80–4.60)	4.40 (4.05–5.00)	*z* = −2.028	.043
Chloride ion	104.00 (100.00–108.25)	107.00 (101.50–112.50)	*z* = −2.126	.034
Venous blood glucose	147.00 (111.25–195.50)	147.25 (123.50–214.00)	*z* = −1.031	.303
Arterial blood glucose	135.35 (109.50–180.17)	150.64 (121.43–192.71)	*z* = −1.401	.161
ALT*	27.00 (15.00–78.25)	50.50 (18.50–167.00)	*z* = −2.288	.022
AST†	44.00 (24.00–108.00)	80.75 (32.50–415.50)	*z* = −2.254	.024
ALP	79.00 (57.50–102.75)	82.00 (62.00–110.00)	*z* = −0.706	.480
albumin	3.30 (2.90–3.80)	3.55 (3.05–3.85)	*z* = −1.263	.207
CK	247.00 (77.00–830.50)	364.50 (136.00–1391.50)	*z* = −1.328	.184
CKMB‡	6.00 (3.00–15.75)	14.00 (5.00–47.00)	*z* = −2.249	.025
Lactic acid	1.60 (1.17–2.75)	2.30 (1.50–5.50)	*z* = −2.816	.005
PaCO2	38.00 (33.00–45.00)	36.50 (33.00–42.50)	*z* = −0.82	.412
pH	7.38 (7.33–7.42)	7.37 (7.28–7.41)	*z* = −1.191	.234
Anion gap	16.00 (13.50–18.75)	16.75 (14.50–19.50)	*z* = −1.337	.181
Bicarbonate	20.50 (18.50–23.00)	19.00 (16.50–22.00)	*z* = −2.154	.031
PT	13.40 (12.32–16.17)	14.10 (12.75–15.75)	*z* = −1.162	.245
APTT	29.50 (25.63–36.28)	31.88 (27.00–46.25)	*z* = −1.731	.083
INR	1.25 (1.10–1.45)	1.25 (1.20–1.45)	*z* = −1.206	.228
Lactate and other laboratory test ratios				
CK and lactate ratio	140.67 (52.36–441.62)	131.74 (65.57–479.48)	*z* = −0.185	.854
Arterial glucose and lactate ratio	79.60 (55.26–112.78)	70.85 (34.13–95.25)	*z* = −1.998	.046
Venous glucose and lactate ratio	84.35 (54.32–129.06)	76.38 (41.13–107.69)	*z* = −2.004	.045
Albumin-to-lactate ratio	2.00 (1.00–2.96)	1.50 (0.63–2.23)	*z* = −2.345	.019
Potassium-to-lactate ratio	2.54 (1.58–3.70)	1.94 (0.95–2.81)	*z* = −2.281	.023
Calcium and lactate ratio	4.94 (3.02–6.91)	3.61 (1.64–5.46)	*z* = −2.576	.010
Chloride-to-lactate ratio	62.81 (37.38–86.67)	48.64 (21.49–70.33)	*z* = −2.587	.010

Due to missing laboratory measurements in some patients in the MIMIC-IV database, the number of observations varies slightly across different variables.Findings from ratio variables should be interpreted as exploratory.

ALP = alkaline phosphatase, ALT = alanine aminotransferase, APTT = activated partial thromboplastin time, AST = aspartate aminotransferase, CK = creatine kinase, CKMB = creatine kinase-myocardial band, INR = International Normalized Ratio, MAP = mean arterial pressure, MIMIC-IV = Medical Information Mart for Intensive Care IV, n = number of individuals, PaCO_2_ = Partial pressure of arterial carbon dioxide, pH = potential of hydrogen, PT = prothrombin time.

* Survival case = 99, death case = 50.

† Survival cases = 103, death cases = 50.

‡ Survival cases = 80, death cases = 42.

### 3.3. Multivariate logistic regression analysis

**Figure 2.**
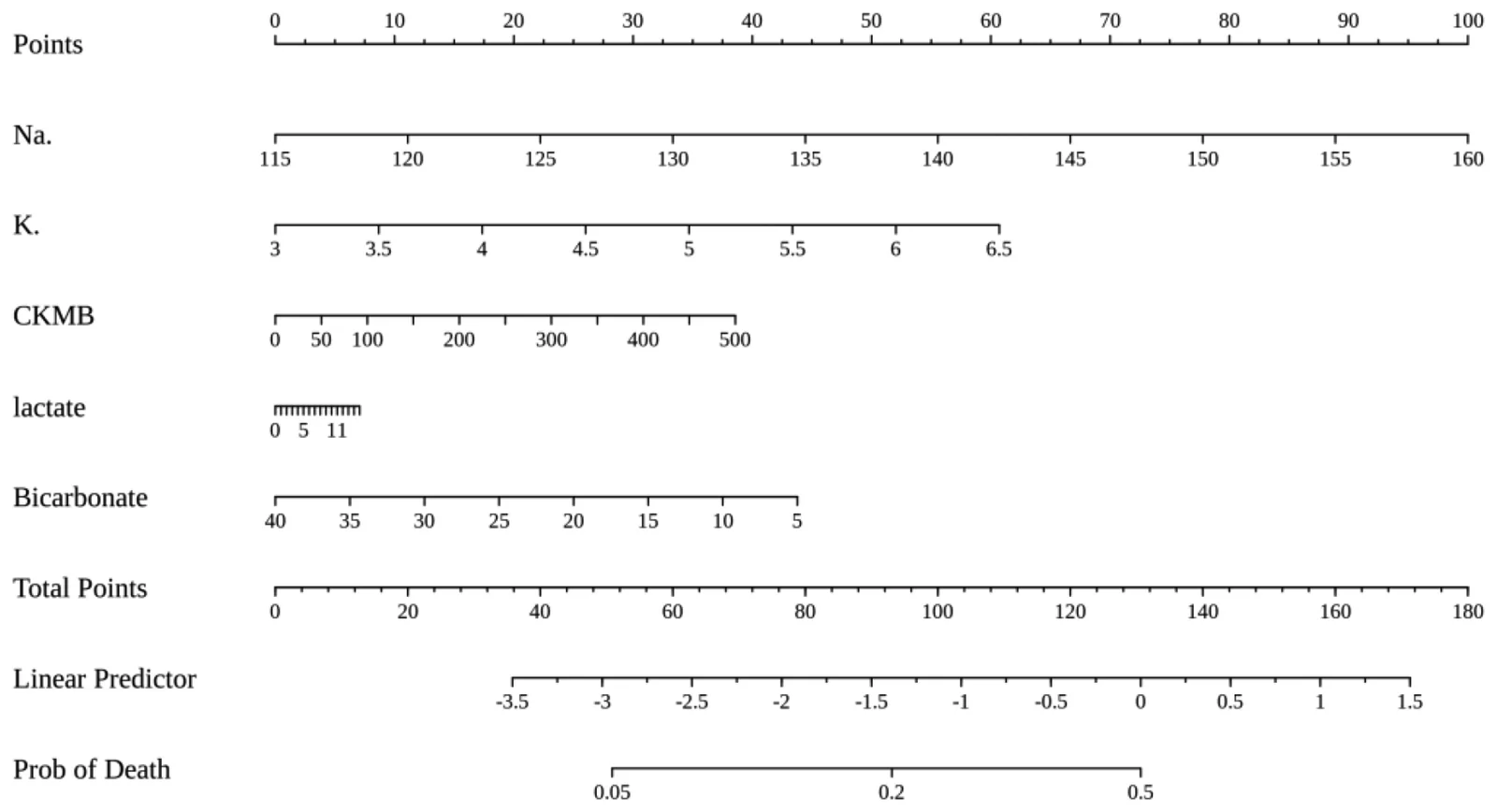
Nomogram for predicting 14-day mortality in ischemic stroke patients. CKMB = creatine kinase-myocardial band, K = potassium, Na = sodium.

**Figure 3.**
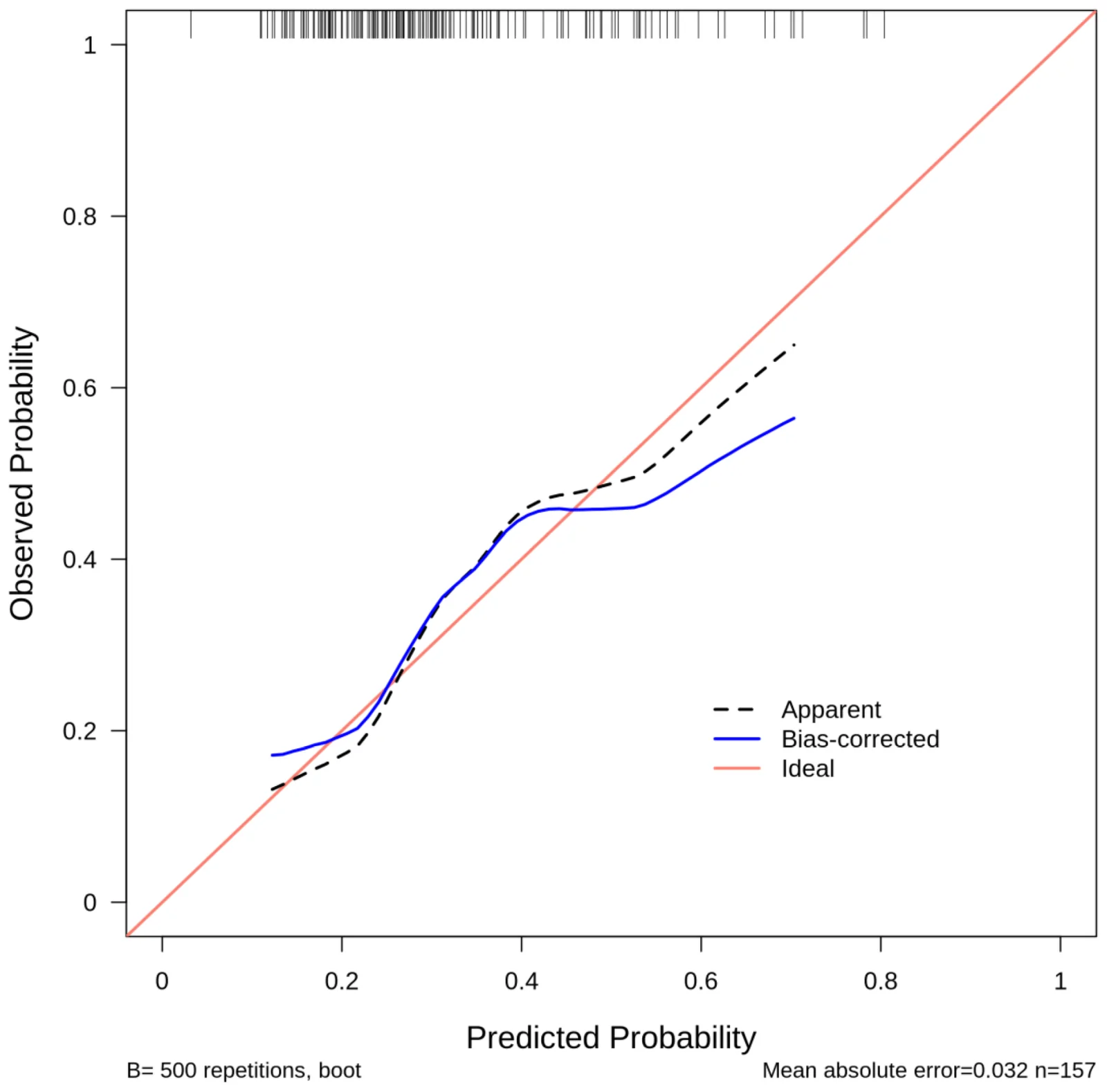
Calibration curve of the multivariable logistic regression model.

Multivariable logistic regression analysis was performed using the candidate variables identified in the univariate analysis and excluded factors with variance inflation factor > 5 to eliminate collinearity. It was found that potassium and sodium ions were independent factors affecting the 14-day survival outcome (*P* < .05), and no statistical differences were found in lactate, CK-myocardial band, and HCO_3_- (*P* > .05). A nomogram prediction model was constructed based on the multivariable logistic regression model to predict 14-day mortality (Fig. 2). The Hosmer–Lemeshow goodness-of-fit test was performed to evaluate the calibration of the model. The test showed no significant difference between the predicted and observed outcomes (*χ*^2^ = 7.122, *P* = .524), indicating that the model had good calibration and no evidence of poor fit. The calibration curve also demonstrated good agreement between the predicted and observed outcomes (Fig. 3). The results of the multivariable logistic regression analysis are presented in Table 4.

**Table 4:** Multivariate logistic regression analysis of single laboratory tests.

	*B*	SE	Wald	Significance	VIF	Tolerance
Module 1						
Na^+^	0.082	0.032	6.754	0.009	1.072	0.082
K^+^	0.627	0.298	4.432	0.035	1.105	0.627
Lactate	0.019	0.083	0.053	0.817	1.345	0.019
CKMB	0.004	0.002	2.488	0.115	1.030	0.004
HCO^3−^	−0.045	0.055	0.679	0.410	1.354	−0.045

Missing data were present for some variables. Descriptive analyses were based on available cases, while multiple imputation was used for regression analyses.

CKMB = creatine kinase-myocardial band, SE = standard error, VIF = variance inflation factor.

### 3.4. ROC analysis of some laboratory test results and exploratory indicators

**Figure 4.**
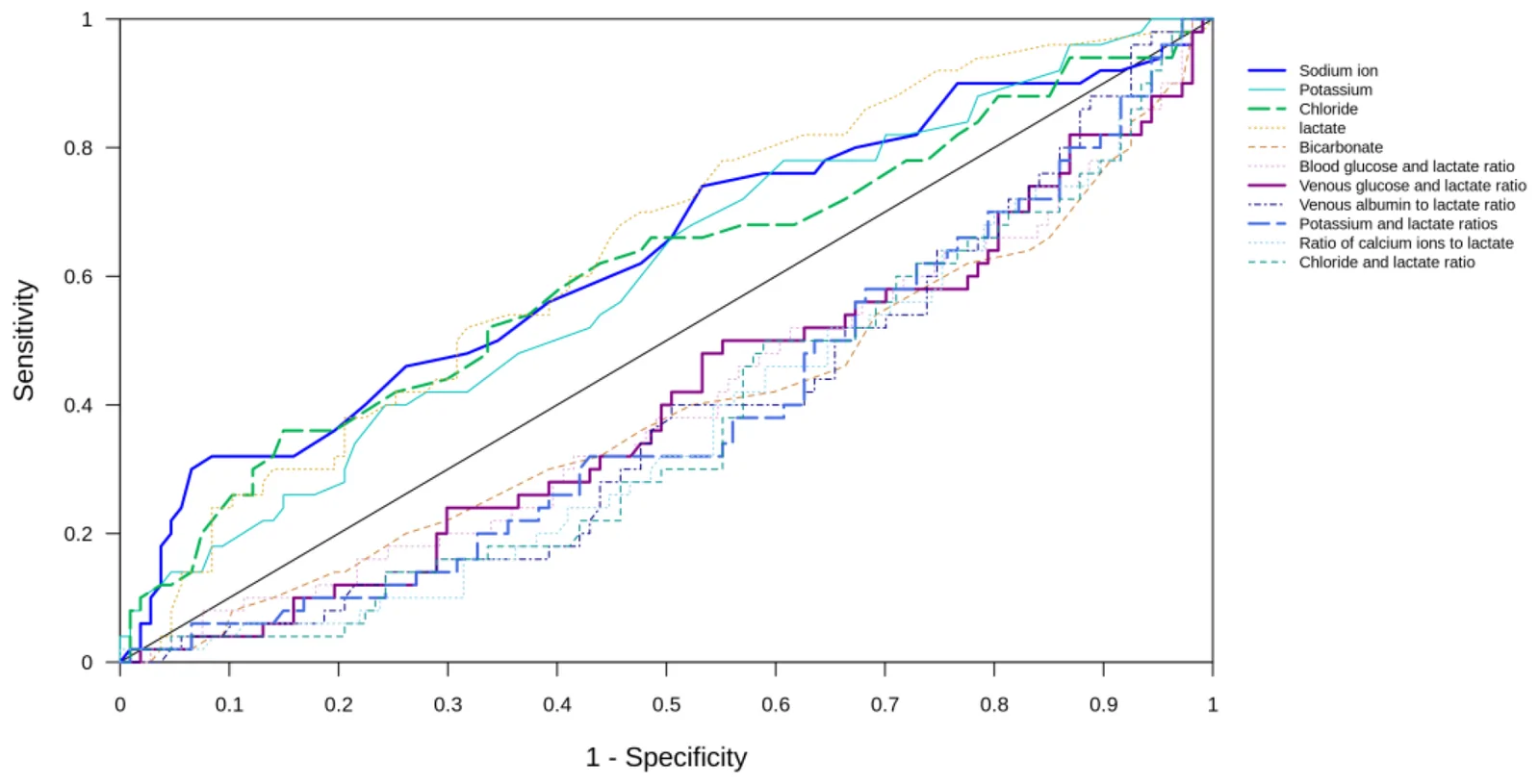
ROC curves of lactate, sodium, potassium, and other exploratory indicators for predicting 14-day mortality. ROC = Receiver operating characteristic.

Table 5 and Figure 4, we analyzed in detail the laboratory test results and exploratory indicators that were found to be statistically significant in the univariate analysis. The ROC analysis showed that potassium ions (AUC = 0.601, 95% CI: 0.506–0.695), sodium ions (AUC = 0.628, 95% CI: 0.531–0.725), chloride ions (AUC = 0.605, 95% CI: 0.507–0.704), and lactate (AUC = 0.637, 95% CI: 0.546–0.728) demonstrated limited discriminative ability for predicting 14-day mortality. These results suggest statistical associations between these indicators and mortality, although their standalone predictive performance is limited. Additionally, the ratio of bicarbonate (AUC = 0.398, 95% CI: 0.300–0.496, *P* = .040), venous glucose and lactate ratio (AUC = 0.402, 95% CI: 0.306–0.497, *P* = .048), venous albumin-to-lactate ratio (AUC = 0.389, 95% CI: 0.296–0.481, *P* = .025), potassium-to-lactate ratio (AUC = 0.39, 95% CI: 0.296–0.484, *P* = .027), calcium and lactate ratio (AUC = 0.372, 95% CI: 0.280–0.463, *P* = .010), and chloride-to-lactate ratio (AUC = 0.374, 95%CI: 0.282–0.466, *P* = .011) were also analyzed. The ROC analysis of several exploratory ratios also showed statistical differences; however, their predictive performance was limited and should be interpreted cautiously.

**Table 5:** ROC analysis of single laboratory tests and exploratory indicators.

Title	AUC	*P*	95% CI	Optimal Threshold	Sensitivity	Specificity	Cutoff
Sodium ion	0.628	.010**	0.531*–*0.725	0.234	0.32	0.914	144.0
Potassium	0.601	.043*	0.506*–*0.695	0.170	0.78	0.390	4.0
Chloride	0.605	.034*	0.507*–*0.704	0.208	0.36	0.848	109.5
lactate	0.637	.006**	0.546*–*0.728	0.228	0.78	0.448	1.45
Bicarbonate	0.398	.040*	0.300*–*0.496	0.019	1.00	0.019	10.00
Blood glucose and lactate ratio	0.403	.051	0.306*–*0.500	0.019	1.00	0.019	10.36
Venous glucose and lactate ratio	0.402	.048*	0.306*–*0.497	0.010	1.00	0.010	9.74
Venous albumin-to-lactate ratio	0.389	.025*	0.296*–*0.481	0.037	0.98	0.057	0.40
Potassium and lactate ratios	0.390	.027*	0.296*–*0.484	0.029	1.00	0.029	0.38
Ratio of calcium ions to lactate	0.372	.010**	0.280*–*0.463	0.020	0.02	1.000	13.17
Chloride and lactate ratio	0.374	.011*	0.282*–*0.466	0.029	1.00	0.029	9.39

Missing data were present for some variables. Descriptive analyses were based on available cases, while multiple imputation was used for regression analyses.Indicators with AUC < 0.5 indicate poor discriminative ability and should be interpreted cautiously.

AUC = area under the curve, CI = confidence interval, ROC = receiver operating characteristic.

* *P* < .05.

** *P* < .01.

### 3.5. Subgroup analysis of the survival and death groups

#### 3.5.1. Pearson correlation analysis in the survival group

In the survival group of this study, there was a correlation between sodium ions and chloride ions, sodium ions and lactate, lactate and arterial blood glucose, lactate and venous blood glucose, arterial blood glucose and venous blood glucose, sodium ions and blood glucose-lactate ratio, and potassium ions and blood glucose-lactate ratio (*P* < .05). There was also a correlation between lactate and various ratios related to lactate (*P* < .05), as shown in Table 6 for details. These correlation analyses were exploratory and aimed to identify potential associations among laboratory indicators within different outcome groups.

**Table 6:** Pearson correlation analysis of laboratory tests and exploratory indicators in the survival group.

		Lactic acid	Sodium ion	Potassium	Calcium	Chloride	Venous blood glucose	Arterial blood glucose	Blood glucose and lactate ratio	Venous albumin–to-lactate ratio	Potassium-to-lactate ratio	Calcium and lactate ratio	Chloride-to-lactate ratio
Sodium ion	*P*	.033											
Potassium	*P*	.094	.253										
Calcium	*P*	.293	.312	.827									
Chloride	*P*	.629	< .001	.521	.290								
Venous blood glucose	*P*	.020	.629	.104	.461	.522							
Arterial blood glucose	*P*	< .001	.606	.772	.863	.555	< .001						
Blood glucose and lactate ratio	*P*	< .001	.010	.045	.799	.237	.051	.164					
Venous albumin-to-lactate ratio	*P*	< .001	.272	.111	.261	.743	.001	< .001	< .001				
Potassium-to-lactate ratio	*P*	< .001	.098	.430	.738	.758	< .001	< .001	< .001	< .001			
Calcium and lactate ratio	*P*	< .001	.199	.204	.114	.597	< .001	< .001	< .001	< .001	< .001		
Chloride-to-lactate ratio	*P*	< .001	.618	.133	.901	.404	< .001	< .001	< .001	< .001	< .001	< .001	

Missing data were present for some variables. Descriptive analyses were based on available cases, while multiple imputation was used for regression analyses.

#### 3.5.2. Pearson correlation analysis in the death group

In the laboratory and exploratory indicators of the death group, there was a correlation between lactate and calcium ions, sodium ions and chloride ions, and arterial blood glucose and venous blood glucose (*P* < .05). There was also a correlation between lactate and various ratios related to lactate (*P* < .05), as shown in Table 7 for details. These findings should be interpreted with caution and require further validation.

**Table 7:** Pearson correlation analysis of laboratory tests and exploratory indicators in the death group.

		Lactic acid	Sodium ion	Potassium	Calcium	Chloride	Venous blood glucose	Arterial blood glucose	Venous glucose and lactate ratio	Venous albumin-to-lactate ratio	Potassium-to-lactate ratio	Calcium and lactate ratio	Chloride-to-lactate ratio
Sodium ion	*P*	.552											
Potassium	*P*	.492	.070										
Calcium	*P*	.047	.974	.622									
Chloride	*P*	.905	< .001	.058	.163								
Venous blood glucose	*P*	.599	.657	.569	.578	.827							
Arterial blood glucose	*P*	.724	.275	.152	.443	.187	< .001						

Missing data were present for some variables. Descriptive analyses were based on available cases, while multiple imputation was used for regression analyses.

## 4. Discussion

### 4.1. Correlation between lactate levels and short-term mortality risk

In this study, univariate analysis found that the initial lactate level was correlated with death within 14 days (*P* < .05); that is, patients with high lactate levels had a higher risk of death within 14 days. Regarding the ROC results, lactic acid showed lower predictive value. However, it’s noteworthy that the optimal cutoff point occurred when sensitivity was 0.78 and specificity was 0.448 (1-specificity was 0.552). At this point, it was closest to the upper left corner (indicating overall optimal performance). The corresponding lactic acid value was 1.45 mmol/L, which is approaching the upper limit of the normal range (0.5–1.7 mmol/L) for fasting whole blood lactic acid. Although lactic acid was significantly associated with mortality in the univariate analysis, it did not show independence in the multivariate analysis, and its predictive ability might be affected by other variables. This result may be affected by many factors. On the one hand, this study adopted strict exclusion criteria and eliminated high-risk diseases that may affect blood test results, such as diabetes, liver and kidney dysfunction, and blood system diseases. This design reduced potential confounding factors and focused the study on the relationship between lactate level and short-term prognosis of stroke. Because this study excluded patients with certain underlying diseases, the relationship between lactate and death was not so obvious, which indirectly indicated that lactate may be related to other underlying diseases. On the other hand, lactate levels may change rapidly in the early stage of hospitalization due to therapeutic interventions (such as fluid replacement or acidosis correction), which may affect the stability of lactate and its independent role in the prognosis of death. Especially in the acute phase, lactate may be interfered with by a variety of internal and external factors, resulting in a large range of changes.^[14,15]^ Therefore, lactic acid as a predictor of death should be interpreted with caution and is more suitable to be used as part of a comprehensive clinical assessment rather than in isolation.

Lactate, as a product of anaerobic metabolism, usually reflects the severity of tissue hypoxia and metabolic disorders and is closely related to the pathological process of ischemic stroke.^[16]^ In brain tissue, lactate is produced and released by normally oxygenated brain cells and by the lactate shuttle system that transports between astrocytes and neurons.^[17]^ Increased lactate levels usually indicate insufficient blood perfusion of brain tissue after ischemia and may interact with factors such as systemic inflammation and cell necrosis, thereby affecting the short-term outcome of stroke patients.^[18,19]^

After a stroke, local lactate production increases significantly, and its increase is usually regarded as a comprehensive manifestation of insufficient brain tissue perfusion and systemic inflammatory response.^[20]^ A large number of experimental studies have clearly confirmed that lactate levels increase significantly after a stroke.^[21–25]^ In the short period after a stroke, the increase in lactate levels is more likely to reflect metabolic stress in the acute phase. However, the complexity of this metabolic disorder may result in the predictive role of lactate in short-term prognosis being lower than its significance in long-term outcomes.^[26,27]^ The study by Geiseler et al^[28]^ further found that L-lactic acid treatment 24 hours and 48 hours after a stroke also has a protective effect on stroke.

Although the initial lactate level in this study was associated with 14-day mortality, it should be noted that lactate is a nonspecific metabolic indicator easily influenced by various factors, including potential infections, tissue hypoxia, occult infections, and early manifestations of sepsis. Existing studies have shown that the predictive efficacy of lactate may be biased by the presence of comorbidities. The lactate levels in patients with spontaneous intracerebral hemorrhage are not only directly affected by brain injury but also may be related to underlying diseases such as hypertension and stroke history. Future research should focus on the relationship between dynamic lactate changes and comorbidities. For example, the lactate clearance rate in sepsis patients can reflect tissue perfusion improvement, while dynamic lactate monitoring in stroke patients may reveal the progression trajectory of neurological damage.

### 4.2. Potassium and sodium levels are independent risk factors for death

This study found that potassium and sodium levels can be used as independent predictors of death (*P* < .05). This result suggests that elevated potassium ion levels may be associated with factors such as electrolyte disorders, cell damage, and abnormal neurological function after a stroke.^[29,30]^ ROC analysis revealed that the AUC values of potassium and sodium ions ranged from 0.60 to 0.70, indicating limited predictive power of single indicators for mortality. However, the optimal cutoff values for potassium and sodium ions were identified as 144 mmol/L and 4 mmol/L, respectively. While the normal human range for sodium is 135 to 145 mmol/L, this value is approaching the upper limit of the normal range. The normal range for potassium is 3.5 to 5.5 mmol/L, suggesting that the study may not have distinguished between patients with hypokalemia and hyperkalemia. Future research should focus on differentiating these patient groups. In a study on the destruction of ion homeostasis caused by traumatic brain injury and ischemic stroke, Martínez–Valverde et al found that the ion characteristics of ischemic stroke patients were high potassium^[31]^, which is similar to the electrolyte disorders found in the death group and the control group in this study. At the same time, the study by Moniruzzaman et al^[32]^ also showed that potassium ion abnormalities are related to stroke. Potassium ions, as a simple and important biomarker, can be used in clinical practice to monitor stroke patients early and provide a reference for therapeutic intervention.

Studies have shown that the mortality rate of ICU patients with serum sodium > 150 mmol/L ranges from 30 to 48%. Even for patients with slightly elevated serum sodium, the mortality rate is higher than that of normal patients (15% vs 30%). ICU-acquired hypernatremia has an independent impact on mortality. Even after adjusting for age, gender, and Apache IV predicted mortality, elevated serum sodium is still associated with an increased risk of death.^[33]^

Electrolyte imbalance is commonly observed in critically ill patients and may reflect systemic physiological instability. In patients with ischemic stroke, disturbances in sodium and potassium levels may be associated with neuroendocrine stress responses, impaired cellular ion homeostasis, and secondary brain injury.^[34,35]^ Abnormal sodium levels may contribute to osmotic imbalance and cerebral edema, while potassium imbalance may affect neuronal excitability and cardiovascular stability.^[36]^ These mechanisms may partially explain the association between electrolyte disturbances and poor outcomes observed in this study.

### 4.3. Lactate and electrolyte metabolism may differ among subgroups

Subgroup analysis found that in the survival group, lactate was correlated with sodium ions, chloride ions, and arteriovenous blood glucose (*P* < .05), indicating that lactate levels may be closely related to electrolyte and metabolic disorders. In the death group, lactate was correlated with calcium ions, sodium ions, and chloride ions (*P* < .05), but not with blood glucose (*P* > .05), which may indicate that the degree of electrolyte metabolism and glucose metabolism disorders was different between the survival group and the death group. Although these results are not yet clearly related to clinical applications, they may provide new clues for a deeper understanding of the role of lactate and electrolytes in the short-term prognosis of stroke patients. This is consistent with other studies reporting the effect of electrolyte disorders on stroke prognosis.^[37–39]^

Previous studies have shown that lactate levels may also be associated with the prognosis of other critical illnesses.^[40–44]^ Yuan Zhong et al^[5]^ analyzed the lactate to albumin ratio of 568 patients with ischemic cerebral infarction based on the MIMIC-IV database. The results showed that the lactate to albumin ratio can be used as an independent predictor of all-cause mortality in patients with ischemic cerebral infarction within 28 days after admission, which is better than using arterial blood lactate or serum albumin alone. Oh et al^[45]^ found that serum lactate level is a predictor of mortality in critically ill patients, and elevated lactate levels are associated with 90-day mortality in patients in the normotensive care unit.

Although lactate did not remain an independent predictor in the multivariable analysis and its discriminative ability was limited (AUC = 0.637), it may still provide useful information regarding the metabolic status of patients with ischemic stroke. In clinical practice, lactate should be interpreted cautiously and is more suitable to be considered as part of a comprehensive clinical assessment rather than used as a standalone prognostic marker. In the management of acute stroke, continuous monitoring of lactate levels can help reveal the evolution of patients’ metabolic state. Future research should focus on developing a multi-indicator joint prediction model that combines lactate, potassium ions, and clinical scores to improve the predictive ability of short-term mortality in stroke patients. In addition, dynamic monitoring of lactate and potassium ions may provide guidance for patients’ individualized intervention strategies and help clinicians manage the condition of stroke patients more accurately. Given the relatively limited sample size and retrospective design, the findings of this study should be considered exploratory and hypothesis-generating rather than confirmatory.

### 4.4. Conclusion

Lactate was associated with short-term mortality in univariate analysis but did not remain an independent predictor after multivariable adjustment. Sodium and potassium levels were independently associated with short-term mortality in patients with ischemic stroke. Electrolyte monitoring should be strengthened. Lactate and electrolyte metabolism may be different in fatal cases. In the future, larger-scale studies should be conducted to combine multiple indicators such as lactate, potassium ions, sodium ions, and underlying diseases to explore their potential for application in stroke management. Clinically, early assessment of electrolyte disturbances alongside lactate levels may provide a simple and accessible approach for preliminary risk stratification in patients with ischemic stroke.

### 4.5. Limitations

This study has the following limitations. First, due to the inherent limitations of retrospective cohort studies, the study cannot clarify the causal relationship between lactate levels and mortality, and there may be selection bias. Second, the data only come from the MIMIC-IV database, and the representativeness of the samples may be insufficient, which needs to be further verified through multicenter studies. Third, this study used stricter exclusion criteria. Because the underlying diseases that may affect the hematological examination were excluded, the effects of lactate and other indicators in the survival group and the death group can be more highlighted, but the effect of the underlying disease on lactate and other indicators cannot be known. Therefore, prospective studies should be carried out in the future, combining the dynamic changes in lactate, potassium ions, and sodium ions, including cases with different underlying diseases, to further evaluate their joint predictive ability for the prognosis of stroke patients. Fourth, the sample size of this study was relatively small, which may affect the stability of the multivariable logistic regression analysis. Therefore, the results should be interpreted with caution.The post hoc subgroup analyses conducted herein are exploratory in nature, aimed at identifying potential interaction effects among distinct subgroups. However, it should be noted that post hoc subgroup analyses may inflate the risk of Type I errors, that is, falsely rejecting the null hypothesis. Consequently, these findings should be considered preliminary and warrant further validation in future studies, preferably through prospective randomized clinical trials.

## Author contributions

**Conceptualization:** YuHao Zhu, Hanghang Song.

**Data curation:** YuHao Zhu, Xinyuan Liu, Hanghang Song.

**Formal analysis:** YuHao Zhu, Xinyuan Liu, Hanghang Song.

**Investigation:** Hanghang Song.

**Methodology:** Hanghang Song.
